# An updated meta-analysis of clinical outcomes, interventions, and treatment approaches in SMuRF vs. SMuRFless: evidence from 2.6 million ACS patients

**DOI:** 10.3389/fcvm.2026.1931296

**Published:** 2026-10-29

**Authors:** Sadia Qazi, Mohammad Hamza Bin Abdul Malik, Muhammad Arham, Anfal Hamza, Muhammad Awais Bin Abdul Malik, Faseeh Haider, Sufyan Shahid, Mishal Khan, Minahil Iqbal, Aleena Sharif, Tahira Nazir, Ayesha Ihsan, Ali Haq

**Affiliations:** 1 College of Medicine, Alfaisal University, Riyadh, Saudi Arabia; 2 Department of Medicine, Nassau University Medical Center, New York City, United States; 3 Department of Cardiology, Sheikh Zayed Medical College, Rahim Yar Khan, Pakistan; 4 Department of Medicine, Allama Iqbal Medical College, Lahore, Pakistan; 5 Department of Medicine, Khawaja Muhammad Safdar Medical College, Sialkot, Pakistan; 6 Department of Medicine, Pakistan Institute of Medical Sciences, Islamabad, Pakistan; 7 Department of Cardiology, Ch Pervaiz Elahi Institute of Cardiology, Multan, Pakistan; 8 Department of Medicine, University Medical and Dental College, Faisalabad, Pakistan; 9 Department of Medicine, The Valley Health System Consortium, Las Vegas, NV, United States

**Keywords:** acute coronary syndrome, angiotensin receptor antagonists / therapeutic use, angiotensin-converting enzyme inhibitors / therapeutic use, SMuRF, SMuRFless

## Abstract

**Background:**

SMuRFless patients with acute coronary syndrome lack traditional modifiable cardiovascular risk factors, however, concerns remain regarding their potentially poorer clinical outcomes.

**Methodology:**

This PRISMA-compliant meta-analysis was registered with PROSPERO (CRD420251021614). A single-arm meta-analysis was conducted to estimate the proportion of SMuRFless patients, while a random-effects frequentist meta-analysis compared SMuRFless vs. SMuRF patients. Statistical analyses were performed using R (version 2025.05.0 + 496; Posit).

**Results:**

Thirty studies comprising 2,650,310 patients were included, of whom 356,716 (13.5%) were SMuRFless. The pooled proportion of SMuRFless patients was 0.11 (95% CI, 0.09–0.14), with substantial heterogeneity (*I*^2^ = 100.0%, *τ*^2^ = 0.6932). Regarding coronary anatomy, SMuRFless patients had less multivessel disease, LCX involvement, and combined RCA/LCX involvement, but more frequent LAD and LMCA involvement than patients with SMuRFs. Despite a lower burden of comorbidities, SMuRFless status was associated with higher in-hospital mortality (RR, 1.68, 95% CI, 1.41–2.01), 30-day mortality (RR, 2.06, 95% CI, 1.16–3.63), cardiogenic shock (RR, 1.46, 95% CI, 1.24–1.70), and cardiac death (RR, 1.61, 95% CI, 1.03–2.50), whereas 1-year, 2-year, 3-year, and 5-year mortality did not differ significantly between groups. Rates of major bleeding, stroke, recurrent myocardial infarction, PCI, and antiplatelet therapy were comparable; however, SMuRFless patients were less likely to undergo CABG (RR, 0.78, 95% CI, 0.61–0.98) or receive ACE inhibitors/ARBs at discharge (RR, 0.87, 95% CI, 0.79–0.96).

**Conclusion:**

SMuRFless patients represent a substantial minority of ACS patients and, despite fewer traditional risk factors, are characterized by distinct coronary anatomy and higher observed short-term adverse event rates.

**Systematic Review Registration:**

https://www.crd.york.ac.uk/PROSPERO/view/CRD420251021614, identifier CRD420251021614.

## 1 Introduction

Acute coronary syndrome (ACS) remains a leading cause of morbidity and mortality worldwide, affecting an estimated 3 million individuals annually (1). ACS encompasses a spectrum of clinical presentations, including unstable angina, non–ST-segment elevation myocardial infarction (NSTEMI), and ST-segment elevation myocardial infarction (STEMI) (2). The pathogenesis of ACS is intrinsically linked to standard modifiable risk factors (SMuRFs), such as hypertension, dyslipidemia, smoking, and diabetes, attributable to approximately 70% of cases (3–5). These risk factors are critical targets for primary and secondary prevention strategies and are emphasized due to their role in risk prediction models, such as the Framingham cardiovascular risk score (6, 7). However, a growing subset of patients presents without these traditional risk factors, challenging conventional diagnostic and therapeutic paradigms (8, 9). This subset of patients was termed “SMuRFless” by Vernon et al. to highlight the need for greater attention to this underexplored group (10).

A nationwide cohort analysis in the U.S. reported that more than 1 in 10 ACS cases occurred in individuals without SMuRFs. An analysis of the Australian CONCORDANCE Registry found that 19% of patients with their first presentation of STEMI were SMuRFless (6, 7, 9). Similarly, varying proportions were reported across different regional studies (11–13). Disparities in the presentation are evident between SMuRFs and SMuRFless, with a higher proportion of SMuRFless patients presenting with STEMI (10, 14). Additionally, the ongoing KiCS-PCI registry found that SMuRFless patients of Japanese ethnicity had significantly higher in-hospital mortality outcomes compared to those with SMuRFs (15). These disparities also extend to management, as guideline-directed medical therapy (GDMT) was not associated with reduced mortality in SMuRFless patients, in contrast to those with SMuRFs (9).

A prior meta-analysis by Kong et al. (2023), including 1,285,722 patients, identified key differences between SMuRF and SMuRFless populations, with 11.56% classified as SMuRFless (16). However, regional studies demonstrate substantial variability in SMuRFless prevalence, and the analysis by Kong et al. is limited by the absence of long-term mortality data. This systematic review and meta-analysis aim to refine the assessment of SMuRFless prevalence with greater granularity and to evaluate associated clinical outcomes, therapeutic interventions, and management strategies in comparison to SMuRF patients.

## 2 Materials and methods

### 2.1 Data source and search strategy

This meta-analysis was conducted following the Preferred Reporting Items for Systematic Review and Meta-Analyses (PRISMA) guidelines (17). A comprehensive literature search was performed using PubMed, Scopus, Clinical Trials, and Cochrane to identify studies comparing SMuRFless ACS patients with those having traditional risk factors (SMuRFs). The search strategy included the following keywords and their MeSH terms: “SMuRFless ACS”, “ACS without traditional risk factors”, “SMuRF vs. SMuRFless”, “Acute Coronary Syndrome”, “Mortality in SMuRFless ACS”, “Cardiogenic shock in ACS”, “Guideline-Directed Medical Therapy (GDMT) disparities”, and “Discharge medications in ACS”. (Supplementary Tables 1, 2). Studies published up to August 2024 were included. Additionally, the references of included articles were reviewed for relevant studies. This meta-analysis was registered in PROSPERO (Registration No. CRD420251021614).

### 2.2 Eligibility criteria

The inclusion and exclusion criteria were structured according to the PICOS (Population, Intervention/Exposure, Comparator, Outcomes, Study design) framework to ensure comparability. **Population:** Adult patients presenting with a first episode of ACS, including STEMI and NSTEMI. **Intervention/Exposure:** Patients presenting without standard modifiable cardiovascular risk factors (SMuRFless). **Comparator:** Patients presenting with at least one standard modifiable risk factor (SMuRF). **Outcomes:** The primary outcomes were the proportion of SMuRFless patients and clinical outcomes including mortality (in-hospital, 30-day, and 1-year), cardiogenic shock, and major bleeding. Secondary outcomes included the utilization of procedural interventions, including coronary artery bypass grafting (CABG) and percutaneous coronary intervention (PCI), as well as GDMT at discharge. **Study Design:** Primary observational studies and randomized controlled trials directly comparing the two groups at their initial ACS presentation. Only articles published in English were eligible. Editorials, commentaries, and other non-primary research articles were excluded. Studies including patients with newly diagnosed or known cardiovascular risk factors outside the SMuRF vs. SMuRFless categorization, or those lacking a direct outcome comparison between the two cohorts, were also excluded.

### 2.3 Data extraction and definitions

An initial screening was conducted using Covidence. Four authors performed the eligibility assessment and data extraction independently (MA, AH, MABAM, SS), with discrepancies resolved in consultation with the senior author (MHBA). Where necessary, means and standard deviations were estimated from medians and interquartile ranges. The following variables were extracted from each study: author, publication year, study design, and sample size. Baseline characteristics included the proportion of SMuRFless and SMuRF patients, median age, body mass index (BMI), and sex distribution. Comorbidities assessed were reduced left ventricular ejection fraction (LVEF < 50%), chronic kidney disease (CKD), peripheral artery disease (PAD), prior heart failure (HF), prior stroke, and family history of coronary artery disease (CAD). Clinical outcomes extracted included in-hospital, 30-day, and 1-year mortality, cardiogenic shock, HF, cardiac death, myocardial infarction (MI), major bleeding, stroke, and cardiac arrest. Procedural and management-related variables included rates of CABG, PCI, and thrombolysis. The utilization of GDMT was recorded, including angiotensin-converting enzyme inhibitors (ACEi) or angiotensin II receptor blockers (ARBs), beta-blockers, statins, aspirin, and P2Y12 inhibitors. Additionally, coronary lesion characteristics such as left main coronary artery (LMCA), left anterior descending artery (LAD), right coronary artery (RCA), and left circumflex artery (LCX) involvement, as well as multivessel disease (MVD) and the presence of atrial fibrillation (AF), were documented. SMuRFs were defined as having at least one standard modifiable cardiovascular risk factor, including hypertension, dyslipidemia, smoking, or diabetes. Patients without any of these risk factors were classified as SMuRFless. All included studies conformed to these definitions. In-hospital mortality was defined as death from any cause during the index admission. Major bleeding was defined as fatal bleeding, and/or symptomatic bleeding in a critical area or organ, and/or bleeding causing a fall in hemoglobin level of 20 g/L or more, or leading to transfusion of two or more units of whole blood or red cells (18).

### 2.4 Quality assessment

The quality of included studies was assessed using the Joanna Briggs Institute (JBI) Critical Appraisal Tool, a widely utilized instrument in prevalence meta-analyses for evaluating methodological rigor and statistical appropriateness (18, 19). Two researchers (MA, SS) independently conducted the quality assessment, with discrepancies resolved through discussion and consultation with a third author (MHBA).

### 2.5 Statistical analysis

All analyses were performed using R (version 2025.05.0 + 496; Posit). The meta package was used for a single-arm proportion meta-analysis, employing the metabin function with a generalized linear mixed model (GLMM) and the Hartung-Knapp adjustment for random-effects confidence intervals, which is recommended for general use (20, 21). The Hartung-Knapp method offers greater accuracy than conventional approaches by imposing constraints on H^2^ when computing variance, thereby enhancing the reliability of effect estimates (22). Subgroup analyses were conducted based on the mean study population age (≥65 years vs. <65 years), study publication year (before vs. after 2020), and economic classification. High-income countries included the United States, United Kingdom, Sweden, Denmark, Australia, Japan, and Singapore. Upper-middle-income countries comprised China, Mexico, Greece, Slovakia, and Poland, while lower-middle-income countries included India and Pakistan. Multinational studies were analyzed separately. Funnel plots were generated to assess publication bias, and sensitivity analysis was conducted using the *metainf()* function. Egger's regression test for small-study effects was performed using the *meta* and *metafor* packages (23). Comparative pairwise analysis between SMuRFless and SMuRF patients was conducted using the DerSimonian and Laird random-effects model, calculating risk ratios (RRs) for dichotomous outcomes and mean differences (MDs) for continuous outcomes (24). A random-effects model was applied to all analyses regardless of heterogeneity, based on the assumption that true intervention effects likely vary. By modeling the distribution of effects, this approach enhances the generalizability of findings beyond studies (25, 26). Heterogeneity was assessed using the *I*^2^ statistic and Cochran's *Q* test, with *I*^2^ values of 25%, 50%, and 75% representing low, moderate, and high heterogeneity, respectively (24). A Cochran's *Q* test *p*-value < 0.10 was considered indicative of significant heterogeneity (27, 28).

## 3 Results

### 3.1 Summary of reviewed articles

A total of 265 records were identified through systematic database searches, comprising 97 records from Scopus, 64 records from PubMed, 52 records from Cochrane CENTRAL, and 2 records from ClinicalTrials.com (as of August 2024). After removing 122 duplicates, 143 records were screened, of which 104 were excluded. Following full-text assessment, 39 reports were reviewed for eligibility, with 9 excluded due to unmet inclusion criteria or disconcordant PICO elements. Ultimately, 30 studies were included in this meta-analysis (Figure 1).

**Figure 1:**
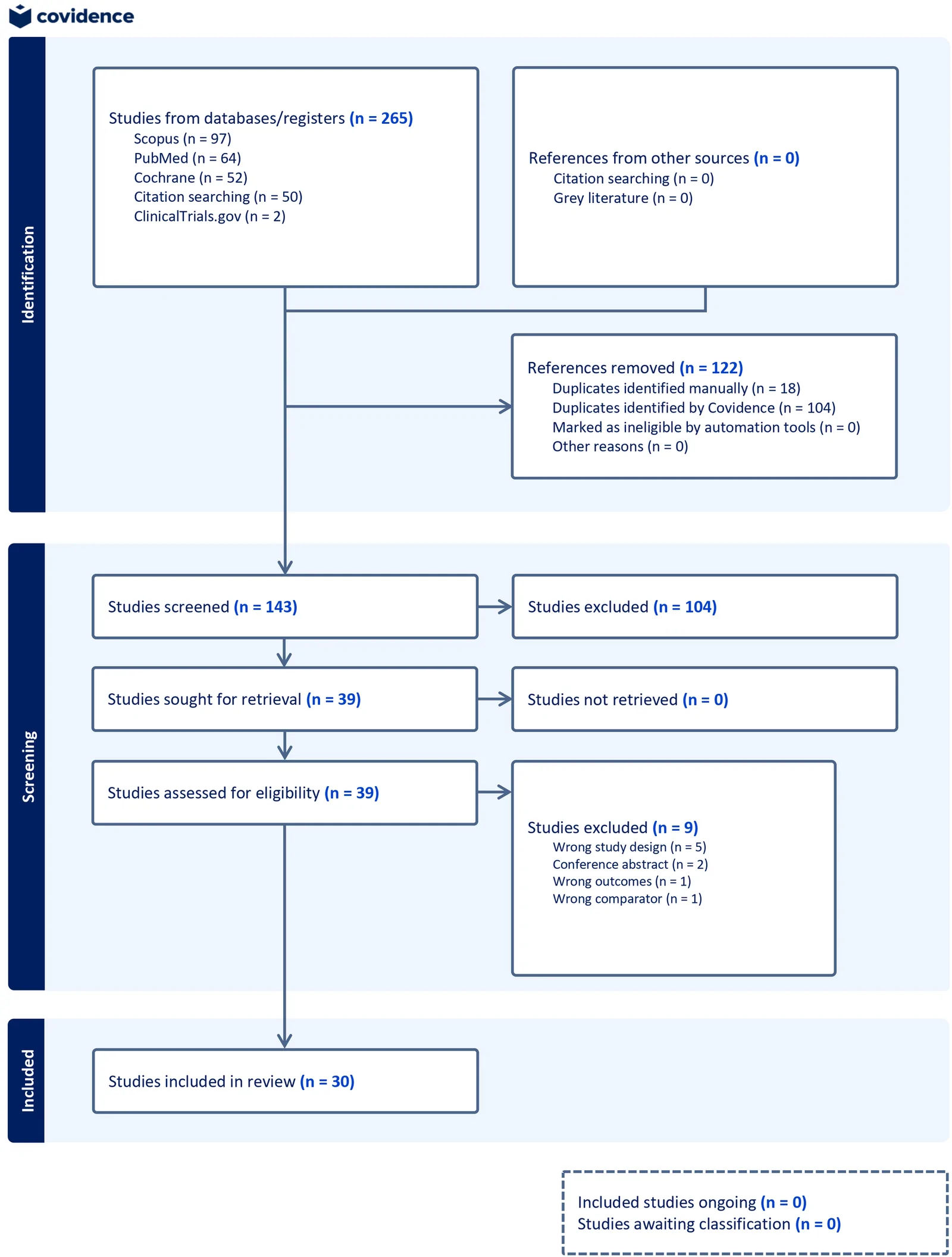
PRISMA (preferred reporting items for systematic reviews and meta-analyses) flow diagram of study selection, detailing the identification, screening, eligibility assessment, and inclusion of studies (17).

This meta-analysis examined ACS patients with and without standard modifiable risk factors (SMuRFless). Among these, two were multinational studies, including the randomized controlled trial by Khot et al., 2003, which encompassed a diverse population from Europe, Mexico, Latin America, the USA, the Middle East, Australia, and Africa (29). The pooled median age was 63.1 years for SMuRFless patients and 58.7 years for SMuRF patients. The quality assessment of the included studies, based on the Joanna Briggs Institute Critical Appraisal Checklist, indicated a low risk of bias for each study (Supplementary Table 3), supporting the reliability of the study findings (Table 1).

**Table 1:** Summary of included studies in the meta-analysis of clinical outcomes, interventions, and treatment approaches in patients With and without standard modifiable cardiovascular risk factors (SMuRFs) presenting With acute coronary syndrome.

Author, Year	Study design	Final Sample Size	N^†^	1/2/3/4 SMuRFs	STEMI^†^	NSTEMI^†^	Median (or mean) Age^†^	BMI^†^
Anderson et al., 2023	Retrospective cohort	8,196	1,458/6,738	1,650/2,230/2,460/398	0/0	1,458/6,738	67/65	-/-
Paul et al., 2023	Retrospective cohort	2,379	605/1,774	-/-/-/-	605/1,774	0/0	55.68/57.44	-/-
Kelly et al., 2023	Retrospective cohort	597	50/547	-/-/-/-	50/547	0/0	42/42	29/26.8
Mazhar et al., 2022	Retrospective cohort	2,046	283/1,763	-/-/-/-	283/1,763	0/0	61/66	27/27
Zao et al., 2023	Retrospective cohort	10,755	1,633/9,142	-/-/-/-	1,633/9,142	0/0	80.29/80.69	-/-
Sia et al., 2022	Retrospective cohort	86,553	811/19,675	4,105/6,237/5,570/3,042	578/11,821	233/7,854	58.2/58.9	24.9/23.3
Sheikh et al., 2023 (14)	Retrospective cohort	15,051	2,267/12,784	5,433/5,005/2,061/285	1,163/5,232	884/5,849	57.8/56.2	-/-
Iwata et al., 2023 (15)	Retrospective cohort	10,523	529/7,246	-/-/-/-	304/4,113	225/3,133	66.9/69.8	23.8/22.4
Jang et al., 2023	Retrospective cohort	474,234	52,242/421,992	-/-/-/-	52,242/421,992	0/0	60.9/62.8	-/-
Moledina et al., 2022	Retrospective cohort	431,615	34,745/396,870	-/-/-/-	34,745/396,870	0/0	63/63	-/-
Saito et al., 2023	Retrospective cohort	115,437	1,777/113,660	-/-/-/-	1,282/81,236	399/28,532	69.8/76.8	-/-
Singh et al., 2024	Retrospective cohort	1,963	126/1,835	-/-/-/-	126/1,835	0/0	67.3/74.5	24/21.6
Suzuki et al., 2022	Retrospective cohort	1,093	64/1,029	-/-/-/-	47/693	17/336	67/71.9	24.2/22.1
Weight et al., 2023	Retrospective cohort	118,177	30,122/88,055	-/-/-/-	30,122/88,055	0/0	63/67	-/-
Figtree et al., 2021 (30)	Retrospective cohort	62,048	9,228/52,820	-/-/-/-	9,228/52,820	0/0	68/69	26.3/25.5
Figtree et al., 2022	Retrospective cohort	2,862	524/2,338	-/-/-/-	524/2,338	0/0	60.7/61	-/-
Vernon et al., 2017 (10)	Retrospective cohort	695	132/404	208/142/52/2	132/404	0/0	64/64	26.9/26.5
Alberty et al., 2017	Retrospective cohort	1,567	90/1,477	321/599/484/73	65/419	8/518	-/-	-/-
Vernon et al., 2019 (31)	Retrospective cohort	3,081	591/2,490	-/-/-/-	591/2,490	0/0	60.4/61.7	28.5/27
Gonzalez et al., 2014	Retrospective cohort	3,447	165/3,282	858/1,165/972/300	121/1,646	44/1,636	-/-	-/-
Roe et al., 2007	Retrospective cohort	74,220	7,755/66,427	22,314/26,329/15,422/2,400	0/0	7,755/66,427	69/72	28.3/26.1
Khot et al., 2003 (29)	Prospective cohort	122,458	22,363/100,095	48,887/34,647/11,868/1,224	14,787/59,360	5,680/29,002	60/67	-/-
Shamaki et al., 2022 (12)	Retrospective cohort	434,111	115,830/318,281	-/-/-/-	115,830/318,281	0/0	67/72	-/-
Kong et al., 2022 (32)	Retrospective cohort	8,680	746/7,934	-/-/-/-	482/4,493	264/3,441	61/57	25.3/25.4
Li et al., 2022	Prospective cohort	16,228	1,918/14,310	-/-/-/-	1,918/14,310	0/0	60.6/64.9	24.5/23.2
Shrestha et al., 2022	Retrospective cohort	433,650	47,495/386,155	-/-/-/-	47,495/386,155	0/0	63.07/62.2	-/-
Papazoglou et al., 2022	Prospective cohort	534	56/478	-/-/-/-	21/201	19/150	63/66	27.9/26.2
Wang et al., 2015	Prospective cohort	13,686	1,984/11,702	-/-/-/-	698/5,451	903/3,167	67/66	-/-
Yamamoto et al., 2021	Retrospective cohort	11,698	479/11,219	-/-/-/-	369/7,943	110/3,276	-/-	-/-
Gladys et al., 2024	Retrospective cohort	182,726	20,665/162,061	59,590/61,881/34,315/6,275	20,665/162,061	0/0	-/-	-/-

† Data are presented in SMuRFless/SMuRF format.

This meta-analysis adhered to the Preferred Reporting Items for Systematic Reviews and Meta-Analyses (PRISMA) guidelines and included 30 studies with a total of 2,650,310 patients. BMI, body mass index; NSTEMI, non-ST-elevation myocardial infarction; SMuRF, standard modifiable risk factor, including hypertension, hyperlipidemia, diabetes mellitus, and smoking status; STEMI, ST-elevation myocardial infarction.

### 3.2 Proportion of SMuRFless ACS patients

A total of 30 studies were included in this systematic review and meta-analysis, encompassing 2,650,310 patients, of whom 356,716 (13.5%) were classified as SMuRFless. The pooled mean proportion of SMuRFless patients was 0.11 (95% CI, 0.09–0.14)**,** with high heterogeneity (*I*^2^ = 100.0%, *τ*^2^ = 0.53). The substantial heterogeneity likely reflects the inclusion of diverse study designs which aim to capture the global proportion of SMuRFless patients. (Figure 2).

**Figure 2:**
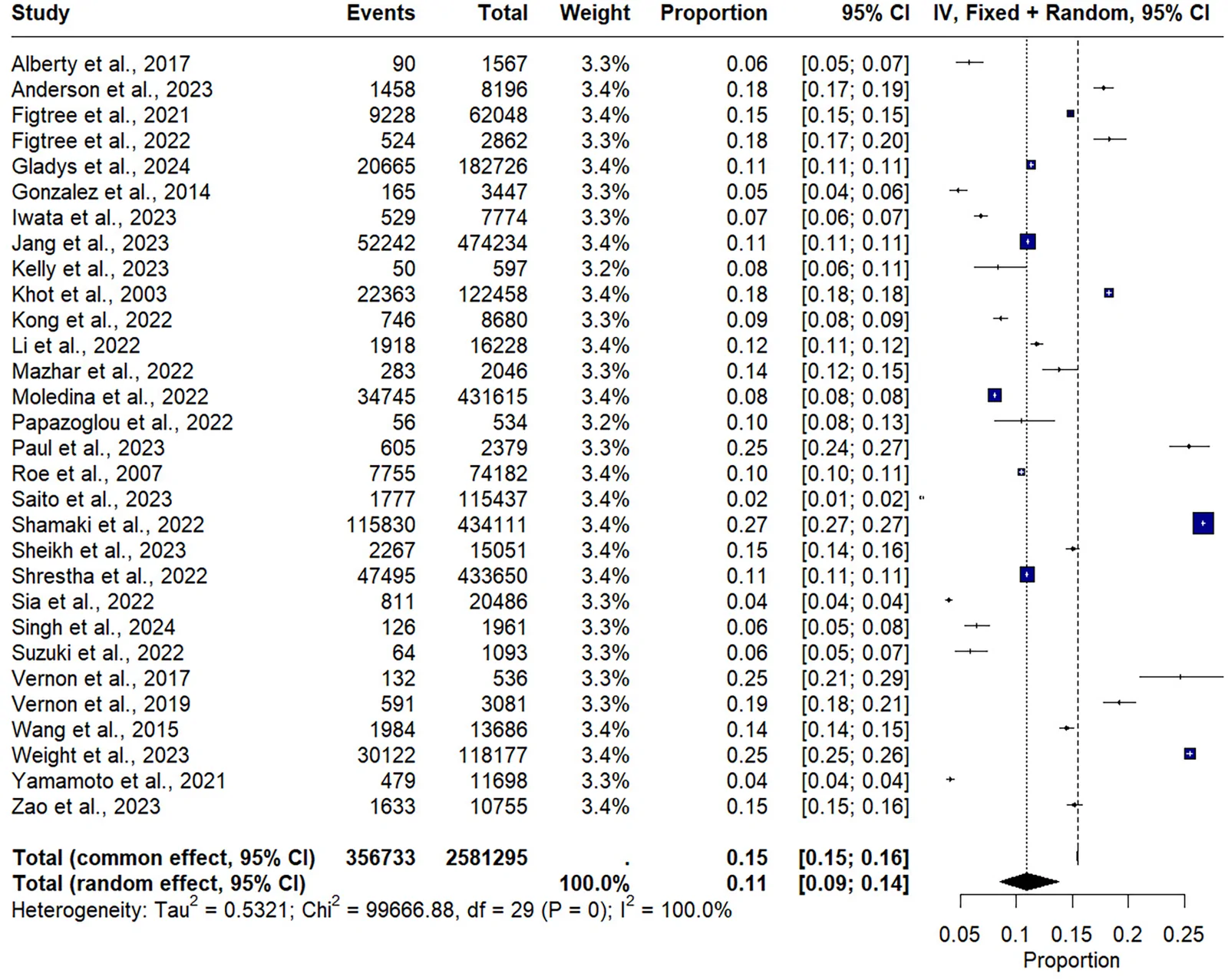
Forest plot depicting the pooled proportion of patients without standard modifiable cardiovascular risk factors across included studies. IV, Inverse Variance; CI, Confidence Interval.

Leave-one-out sensitivity analysis showed consistent pooled estimates with persistent high heterogeneity (*I*^2^ = 100.0%), indicating that no single study significantly influenced the overall results. The funnel plot (Figure 3) shows an asymmetrical distribution of studies. However, Egger's regression test did not indicate significant small-study effects (*z* = −1.5406, *p* = 0.1234), reducing concerns about major bias.

**Figure 3:**
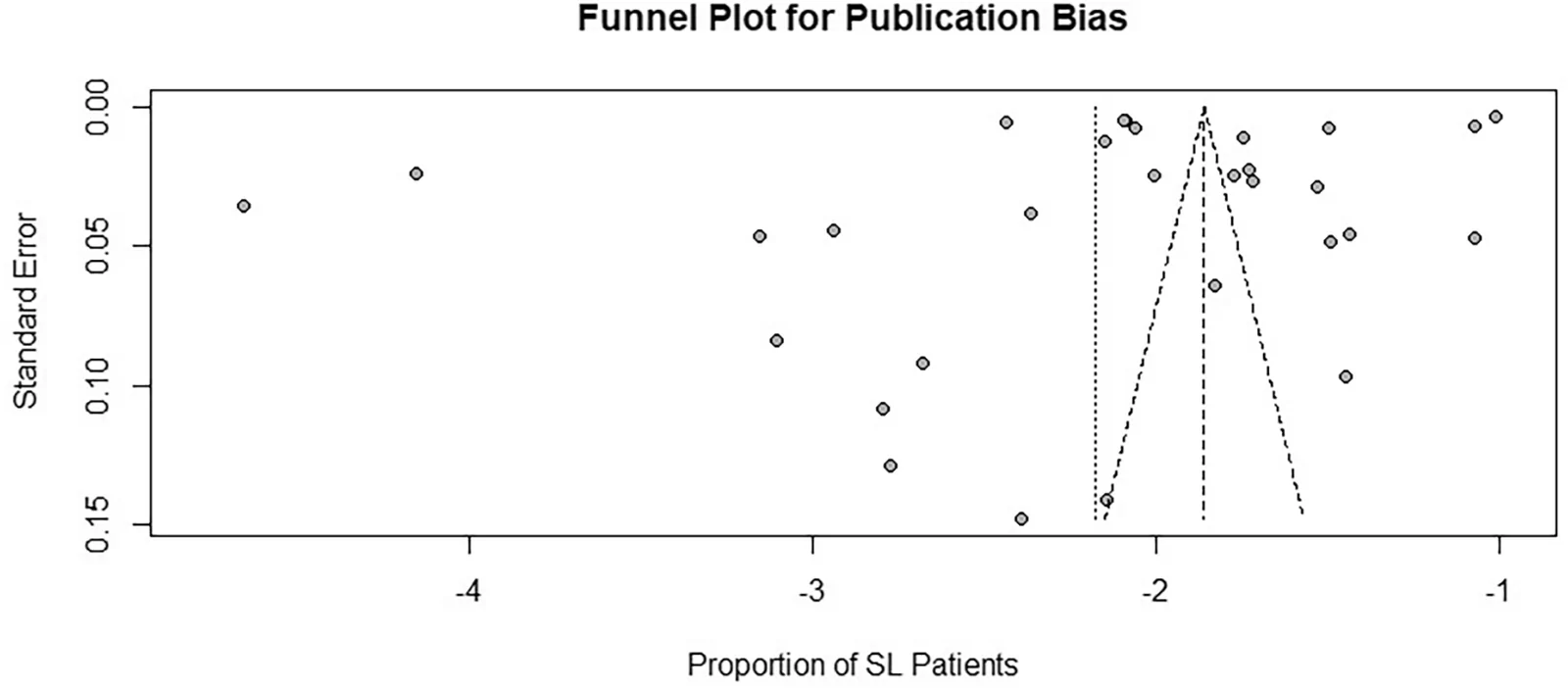
Funnel plot assessing publication bias in the included studies.

### 3.3 Subgroup analysis of proportion of SMuRFless ACS patients

In subgroup analyses based on age, the proportion of SMuRFless patients was 0.11 (95% CI, 0.08–0.16) in studies with a mean age ≥ 65 years (*I*^2^ = 100.00%, *τ*^2^ = 0.65) and 0.11 (95% CI, 0.07–0.17) in studies with a mean age < 65 years (*I*^2^ = 99.90%, *τ*^2^ = 0.77). No significant difference was observed between these subgroups (*χ*^2^ = 0.01, df = 1). The proportion of SMuRFless patients remained consistent over time, with estimates of 0.10 (95% CI, 0.07–0.14; *I*^2^ = 100.00%, *τ*^2^ = 0.81) in studies published after 2020 and 0.11 (95% CI, 0.08–0.16; *I*^2^ = 99.80%, *τ*^2^ = 0.35) in studies published before 2020. There was no significant difference between these time periods (*χ*^2^ = 0.27, df = 1). The proportion of SMuRFless patients varied by income level, as classified by the World Bank 2024–2025 income classification. In high-income countries, including the United States, United Kingdom, Sweden, Denmark, Australia, Japan, and Singapore, the proportion was 0.09 (95% CI, 0.06–0.13; *I*^2^ = 100.00%, *τ*^2^ = 0.83). Upper-middle-income countries such as China, Mexico, Greece, Slovakia, and Poland reported a similar proportion of 0.10 (95% CI, 0.07–0.14; *I*^2^ = 98.70%, *τ*^2^ = 0.22). However, lower-middle-income countries, specifically India and Pakistan, exhibited a notably higher proportion of 0.20 (95% CI, 0.13–0.28; *I*^2^ = 99.40%, *τ*^2^ = 0.11). Multinational studies reported a proportion of 0.16 (95% CI, 0.14–0.19; *I*^2^ = 99.20%, *τ*^2^ = 0.02). A significant difference was observed across these income-based subgroups (*χ*^2^ = 15.72, df = 3).

### 3.4 Clinical characteristics and presentation: SMURF vs. SMuRFless

Among patients with ACS, the proportion of males was similar between those with and without standard modifiable risk factors [SMuRFless vs. SMuRF, relative risk (RR) 1.00, 95% CI, 0.94–1.07]. Left ventricular dysfunction was comparable between groups (RR, 0.94, 95% CI, 0.82–1.09). The prevalence of comorbidities was significantly lower in SMuRFless patients, including CKD (RR, 0.58, 95% CI, 0.37–0.91), prior stroke (RR, 0.61, 95% CI, 0.46–0.82), prior HF (RR, 0.63, 95% CI, 0.43–0.93), and PAD (RR, 0.50, 95% CI, 0.41–0.61). The family history of CAD was also comparable (RR, 0.80, 95% CI, 0.64–1.00) (Table 2).

**Table 2:** Comparison of clinical characteristics between acute coronary syndrome patients with and without standard modifiable risk factors.

Clinical characteristics	*N*	SMuRFless		SMuRF		RR/MD, 95% CI	*I* ^2^
		Event	Totals	Events	Total		
Demographics							
Gender (male)	20	76,422	104,301	598,935	846,193	RR: 1.00 [CI: 0.94; 1.07]	98.0%
Vessel distribution							
MVD^†^	13	7,457	24,613	115,428	272,022	RR: 0.83 [CI: 0.71; 0.95]	98.6%
LAD^†^	9	13,381	33,171	92,137	242,946	RR: 1.11 [CI: 1.07; 1.16]	71.3%
LMCA^†^	4	169	11,058	977	73,747	RR: 1.50 [CI: 1.24; 1.81]	0.0%
LAD/LMCA	11	14,788	35,477	102,741	363,852	RR: 1.38 [CI: 0.88; 2.14]	99.9%
RCA	9	11,308	34,898	132,069	356,059	RR: 0.90 [CI: 0.80; 1.01]	85.0%
LCX^†^	8	1,450	14,233	31,632	193,998	RR: 0.89 [CI: 0.85; 0.93]	0.0%
RCA/LCX^†^	10	12,966	35,427	166,884	363,305	RR: 0.89 [CI: 0.83; 0.96]	82.1%
Others/unspecified	4	1,759	11,170	9,933	65,018	RR: 1.02 [CI: 0.97; 1.06]	0.0%
Co-morbid and other risk factors							
CKD^†^	15	16,612	293,958	179,548	18,26,615	RR: 0.58 [CI: 0.37; 0.91]	99.4%
Prior Stroke^†^	16	3,897	254,463	36,637	13,75,503	RR: 0.61 [CI: 0.46; 0.82]	98.3%
Prior HF^†^	14	17,072	143,186	177,000	10,97,247	RR: 0.63 [CI: 0.43; 0.93]	98.1%
PAD^†^	13	9,855	296,674	102,201	17,17,773	RR: 0.50 [CI: 0.41; 0.61]	95.4%
Family History of CAD	15	19,493	214,534	134,487	955,355	RR: 0.80 [CI: 0.64; 1.00]	98.8%
LVEF (<50%)	7	5,254	16,212	42,508	116,686	RR: 0.94 [CI: 0.82; 1.09]	88.0%

† indicates statistically significant findings.

This meta-analysis adhered to the Preferred Reporting Items for Systematic Reviews and Meta-Analyses (PRISMA) guidelines and included 30 studies with a total of 2,650,310 patients. N, number of studies; MVD, multivessel disease; LAD, left anterior descending artery; LMCA, left main coronary artery; LAD/LMCA, left anterior descending artery/left main coronary artery; RCA, right coronary artery; LCX, left circumflex artery; RCA/LCX, right coronary artery/left circumflex artery; LVEF, left ventricular ejection fraction; CKD, chronic kidney disease; PAD, peripheral artery disease; CAD, coronary artery disease; RR, relative risk; MD, mean difference.

Compared with patients with SMuRFs, SMuRFless patients had a lower likelihood of multivessel disease (MVD; RR, 0.83; 95% CI, 0.71–0.95; *I*^2^ = 98.6%), left circumflex artery (LCX) involvement (RR, 0.89; 95% CI, 0.85–0.93; *I*^2^ = 0%), and combined RCA/LCX involvement (RR, 0.89; 95% CI, 0.83–0.96; *I*^2^ = 82.1%). In contrast, LAD (RR, 1.11; 95% CI, 1.07–1.16; *I*^2^ = 71.3%) and LMCA involvement (RR, 1.50; 95% CI, 1.24–1.81; *I*^2^ = 0%) were more frequent among SMuRFless patients (Figure 4).

**Figure 4:**
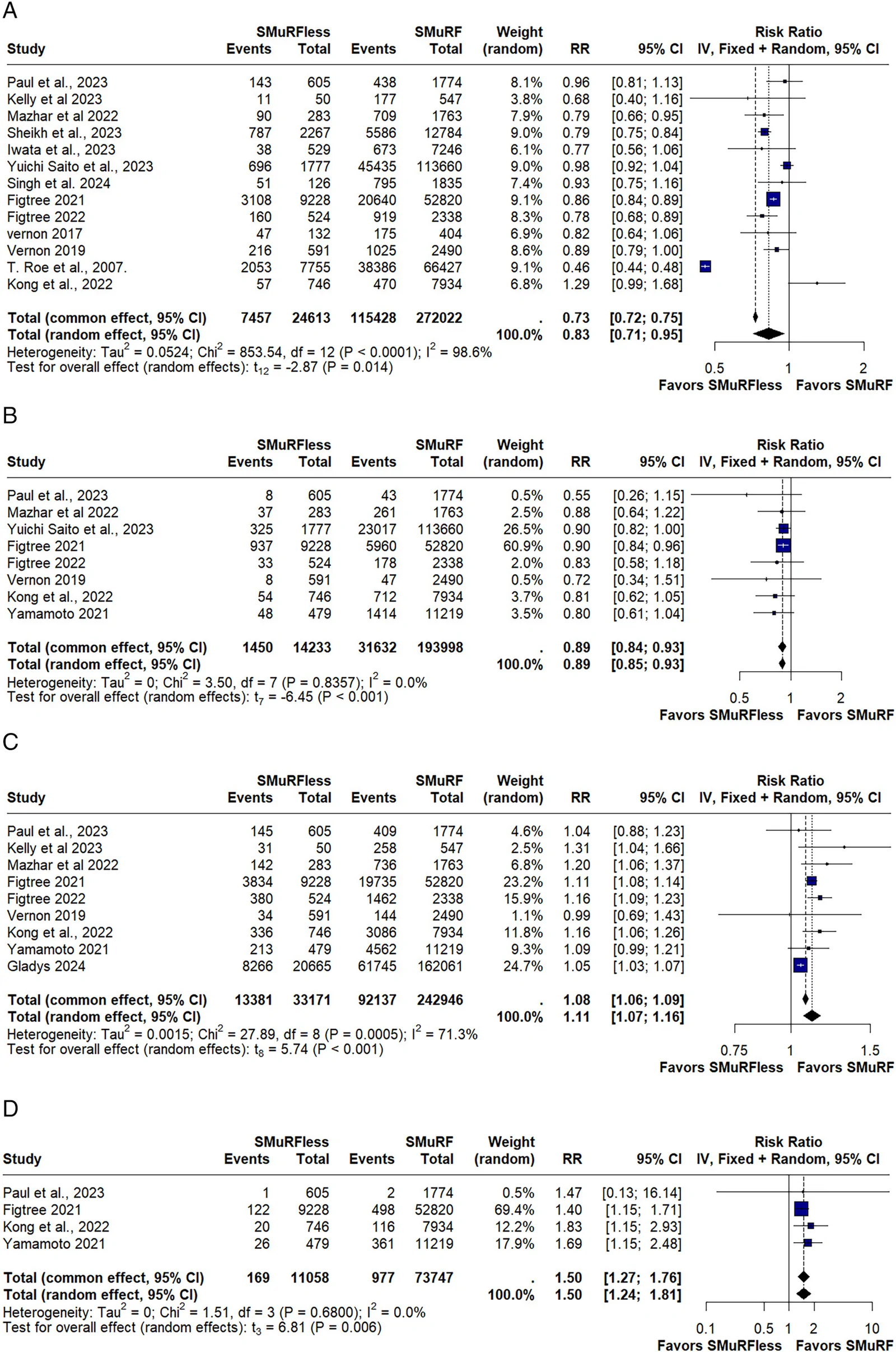
Forest plot of coronary vessel distribution in patients without standard modifiable cardiovascular risk factors (SMuRFless) vs. those with SMuRFs. **(A)** Multivessel disease. **(B)** Left circumflex artery. **(C)** Left Anterior Descending Artery. **(D)** Left Main Coronary Artery. IV, Inverse Variance; CI, Confidence Interval.

### 3.5 Clinical management and outcomes: SMURF vs. SMRUF-Less

Among patients with ACS (Table 3), hospital mortality was significantly higher in the SMuRFless group than in the SMuRF group (RR, 1.68, 95% CI, 1.41–2.01; *I*^2^ = 98.9%). However, subgroup analysis according to STEMI vs. NSTEMI classification showed no significant difference, and there was no evidence of publication bias (*p* = 0.74). Similarly, 30-day mortality was significantly higher in SMuRFless patients (RR, 2.06, 95% CI, 1.16–3.63; *I*^2^ = 91.5%), while subgroup analysis by STEMI vs. NSTEMI classification again showed no significant difference. No evidence of publication bias was observed for 30-day mortality (*p* = 0.4907). In contrast, no significant differences between SMuRFless and SMuRF patients were observed for 1-year, 2-year, 3-year, or 5-year mortality. The incidence of major bleeding (RR, 1.12, 95% CI, 0.91–1.39), stroke (RR, 1.06, 95% CI, 0.81–1.38), and recurrent myocardial infarction (RR, 0.83, 95% CI, 0.65–1.07) was non-significant and comparable between groups. SMuRFless patients were less likely to undergo CABG (RR, 0.78, 95% CI, 0.61–0.98), while PCI and thrombolysis rates were similar. At discharge, SMuRFless patients were less frequently prescribed ACEi/ARBs (RR, 0.87, 95% CI, 0.79–0.96). Use of beta-blockers, statins, aspirin, and P2Y12 inhibitors did not differ significantly between groups.

**Table 3:** Comparison of management and outcomes between acute coronary syndrome patients with and without standard modifiable risk factors.

Clinical characteristics	*N*	SMuRFless		SMuRF		(RR/MD, 95% CI)	*I* ^2^
		Events	Total	Events	Total		
Outcomes							
Hospital Mortality^†^	20	55,185	330,187	160,541	20,80,256	RR: 1.68 [CI: 1.41; 2.01]	98.9%
30-day Mortality^†^	11	2,849	86,300	12,662	676,832	RR: 2.06 [CI: 1.16; 3.63]	91.5%
1-Year Mortality	8	4,015	32,478	20,925	234,590	RR: 1.24 [CI: 0.67; 2.29]	97.2%
2-Year Mortality	3	1,553	9,317	7,744	54,243	RR: 1.46 [CI: 0.45; 4.72]	54.2%
3 Year Morality	3	1,686	9,990	9,344	65,802	RR: 1.35 [CI: 0.69; 2.65]	92.7%
5-Year Mortality	3	2,960	10,207	17,853	56,306	RR: 0.79 [CI: 0.08; 7.72]	99.5%
Atrial Fibrillation	12	12,192	123,891	111,336	10,84,475	RR: 0.88 [CI: 0.69; 1.12]	94.1%
Cardiogenic Shock^†^	13	38,798	222,173	145,691	13,79,495	RR: 1.46 [CI: 1.24; 1.70]	98.4%
Cardiac Death^†^	4	2,114	31,938	6,513	109,698	RR: 1.61 [CI: 1.03; 2.50]	95.3%
Heart Failure	16	16,076	192,996	100,181	10,16,430	RR: 0.90 [CI: 0.74; 1.09]	95.8%
Major Bleeding	12	6,864	137,303	48,518	11,47,172	RR: 1.12 [CI: 0.91; 1.39]	96.4%
Recurrent MI	13	3,639	154,738	29,015	10,70,995	RR: 0.83 [CI: 0.65; 1.07]	73.6%
Stroke	11	1,561	60,306	11,919	584,831	RR: 1.06 [CI: 0.81; 1.38]	84.5%
Interventions							
CABG^†^	12	4,755	138,788	62,746	10,57,504	RR: 0.78 [CI: 0.61; 0.98]	95.2%
PCI	21	112,504	217,931	972,639	17,09,341	RR: 1.03 [CI: 0.90; 1.18]	99.8%
Thrombolysis	9	10,584	71,133	60,074	5,78,048	RR: 0.91 [CI: 0.83; 1.00]	88.3%
Discharge medications							
ACEi/ARBs^†^	16	40,992	59,583	2,19,811	3,08,004	RR: 0.87 [CI: 0.79; 0.96]	97.8%
Beta Blocker	16	48,200	59,583	2,52,484	3,08,004	RR: 0.95 [CI: 0.88; 1.02]	93.3%
Statins	16	47,993	59,583	2,61,209	3,08,004	RR: 0.94 [CI: 0.87; 1.02]	94.6%
Aspirin	14	48,038	51,702	2,23,017	2,39,742	RR: 1.04 [CI: 0.94; 1.15]	95.7%
P2Y12 Inhibitors	10	45,145	54,920	2,21,743	2,73,118	RR: 1.00 [CI: 0.88; 1.13]	98.3%

† indicates statistically significant findings.

This meta-analysis adhered to the Preferred Reporting Items for Systematic Reviews and Meta-Analyses (PRISMA) guidelines and included 30 studies with a total of 2,650,310 patients. N, number of studies; CABG, coronary artery bypass graft; PCI, percutaneous coronary intervention; ACEi, angiotensin-converting enzyme inhibitor; ARB, angiotensin receptor blocker; MI, myocardial infarction; SMuRF, standard modifiable risk factor; RR, risk ratio.

## 4 Discussion

This systematic review and meta-analysis found significant disparities in clinical outcomes, procedural interventions and pharmacological treatment between SMuRFless and SMuRF ACS groups. SMuRFless status was associated with significantly higher rates of in-hospital mortality, 30-day mortality, cardiogenic shock, and cardiac death, despite broadly comparable rates of revascularization and use of most evidence-based secondary prevention therapies. However, ACE inhibitor/ARB therapy was modestly underutilized among SMuRFless patients. SMuRFless individuals were less likely to have comorbidities such as CKD, PAD, prior HF, and prior stroke.

When we compare our findings to previous meta-analyses, several consistent trends and emerging discrepancies were observed. Similar to the previous meta-analysis (9), SMuRFless patients had significantly higher observed in-hospital and 30-day mortality and a higher incidence of cardiogenic shock, compared with SMuRF patients. These findings do not support the assumption that the absence of conventional risk factors is necessarily associated with better short-term outcomes in ACS. Additionally, our findings contradict previous reports indicating that SMuRFless patients are less likely to receive GDMT (9, 15, 16, 33). Although earlier studies reported inconsistent differences in CABG utilization across cohorts, our updated analysis suggests that SMuRFless patients undergo CABG slightly less frequently than SMuRF patients across multiple healthcare settings, despite broadly comparable overall revascularization rates (16). Current guidelines from the American College of Cardiology (ACC), the American Heart Association (AHA) (2016), and the European Society of Cardiology (ESC) (2023) recommend risk stratification and personalized approaches for treating ACS patients (34, 35). These guidelines strongly recommend immediate revascularization strategies, including PCI and CABG, depending on the severity of CAD as assessed by Society for Thoracic Surgeons (STS) risk score or the European System for Cardiac Operative Risk Evaluation (EuroSCORE) II (35, 36). However, there is a notable gap in these guidelines regarding specific recommendations tailored to SMuRFless patients, who, as demonstrated in our study, have distinct clinical profiles and worse outcomes despite the lower risk status. The absence of tailored guidelines for this subgroup highlights the urgent need for updates that incorporate emerging evidence from studies like ours. Additionally, both the ACC/AHA and ESC advocate for strict adherence to GDMT, including dual-antiplatelet therapy (DAPT), beta-blockers, statins, and renin-angiotensin-aldosterone system inhibitors, to reduce mortality and prevent recurrent events in all ACS patients (35, 37). This presumed association between suboptimal adherence to GDMT and high mortality in SMuRFless patients has been challenged by emerging evidence. A recent study by Gong et al, found that GDMT was not associated with lowering mortality in SmuRFless patients, unlike the association in SMuRF patients (9). A national registry analysis from the Chinese Cardiovascular Association Database–Chest Pain Center similarly demonstrated that the higher observed risk in SMuRFless patients persisted after adjustment for receipt of GDMT, although residual confounding cannot be excluded (38). This observation raises the possibility that adverse outcomes associated with SMuRFless ACS may involve mechanisms not fully captured by conventional secondary-prevention strategies. Additionally, much of the benefit of statins and RAAS inhibitors comes from lowering LDL cholesterol and blood pressure, and these risk factors are, by definition, absent or below treatment thresholds in this group (39). Contemporary evidence remains limited, with no RCT specifically evaluating GDMT in SMuRFless patients. Nevertheless, adherence to current GDMT remains the most pragmatic approach; given the poorly understood pathophysiological mechanisms underlying SMuRFless presentations, existing evidence-based recommendations should continue to be followed until tailored management strategies and dedicated guidelines emerge (35, 37, 40). Meanwhile, future trials should explore biomarker-, imaging-, or genotype-guided approaches to identify distinct SMuRFless phenotypes that may benefit from targeted therapies.

The Chinese Cardiovascular Association Database–Chest Pain Center analysis was also consistent with our findings, demonstrating higher early mortality among SMuRFless patients, while adjusted long-term mortality did not differ significantly between SMuRFless and SMuRF groups (38). The higher early mortality observed among SMuRFless patients may reflect several potential mechanisms. One potential contributor to the observed early mortality difference may be the greater proportion of SMuRFless patients presenting with STEMI; however, subgroup analyses did not demonstrate a significant difference according to STEMI vs. NSTEMI classification (16, 41). Second, our study found that SMuRFless patients demonstrated greater involvement of the left main and left anterior descending arteries as the culprit vessels, which supply larger myocardial territories and, as a result, have a much worse prognosis (41, 42). Other recent studies, including two extensive registry-based studies, CONCORDANCE [Cooperative National Registry of Acute Coronary Syndrome Care] and SWEDEHEART (30, 31), suggest that differences in the contribution of pathophysiologic pathways in individuals with heightened susceptibility to coronary atherosclerosis irrespective of comorbid conditions may be more impactful in the cellular environment of the LAD artery (43). Whether this relates to differences in embryologic origin or differences in biomechanical forces warrants further investigation (43–45). Our findings are also consistent with the culprit-vessel literature, which suggests that the excess mortality associated with LAD infarction is largely concentrated within the first month after MI (46). It also matches SWEDEHEART data showing that SMuRF-less NSTEMI patients who survive the first 30 days have lower long-term mortality than patients with risk factors (30). Third, arrhythmias may also play a key role in the increased mortality observed in this group. The higher incidence of atrial fibrillation found in this group has been frequently associated with severe LV damage and heart failure (47). In fact, the development of atrial fibrillation in the setting of acute MI is an independent predictor of all-cause mortality, irrespective of the treatment given (48, 49). However, our pooled analysis did not demonstrate a significant difference in atrial fibrillation between SMuRFless and SMuRF patients (RR, 0.88, 95% CI, 0.69–1.12), suggesting that atrial fibrillation itself is unlikely to explain the observed difference in mortality between the two groups. Key observation, however, is that SMuRFless patients experience worse early outcomes (50). The literature addressing this phenomenon remains limited and may reflect factors such as missed diagnoses, under-recognition of disease severity, and distinct underlying pathophysiological mechanisms.

Although current guidelines lack treatment and therapeutic approaches specific to ACS patients without traditional risk factors, a clinical pathway navigating diagnostic, procedural and treatment approaches to CAD patients without standard modifiable risk factors has been developed by an international, multidisciplinary team in an effort to improve outcomes through enhanced adherence to evidence-based secondary prevention and additional diagnosis of observed modifiable risk factors (51). A proposed four-step pathway by Figtree et al, which has not yet been validated in prospective studies, outlines the evaluation and management of SMuRFless CAD. It includes confirmation of SMuRFless status, guideline-based secondary prevention with DAPT, ACEI/ARB therapy, beta-blockers when indicated, high-intensity statins, and cardiac rehabilitation, followed by additional investigations to identify potential underlying mechanisms. These may include metabolic, lipid, inflammatory, thrombotic, lifestyle, and other targeted assessments. This proposed pathway should therefore be distinguished from established ACC/AHA and ESC evidence-based recommendations, and its clinical utility requires evaluation in prospective registries and clinical trials.

RCTs and clinical guidelines need to recognize the significant proportion of SMuRFless patients and provide specific recommendations regarding PCI and GDMT for patients with SMuRFless presentations. Future research should focus on genetic analyses, mendelian randomization studies, and the identification of specific polymorphisms responsible for atherogenesis that leads to ACS in SMuRFless individuals to develop clinically relevant polygenic risk scores. Moreover, because post-transcriptional modifications may also underlie ACS without SMuRFs, further research building on microRNAs and proteomic/metabolomic biomarkers, such as ceramides, acylcarnitines, lipoproteins, adiponectin, and galectin-3, is warranted to elucidate additional pathogenetic aspects. Additionally, advances in multi-omic technologies and machine learning provide new platforms to inform the development of blood-based biomarkers and novel therapies (31, 52). Whilst coronary artery calcium scores have the potential for screening for subclinical disease, guidelines do not currently recommend this in patients classified as low risk based on the absence of SMuRFs (40, 53). However, this could be a potential non-invasive approach to test the accuracy of emerging tools, such as polygenic risk scores and novel biomarkers, that may address the unmet needs of SMuRFless CAD patients (54). Ultimately, given the effectiveness of existing therapies in halting the progression of early atherosclerosis, novel imaging modalities may contribute to the early and comprehensive assessment of plaques, providing all individuals with the opportunity to prevent future heart attacks. Prospective and randomized evidence remains limited. Future studies should use standardized SMuRF definitions, rigorous confirmation of SMuRFless status, and systematic assessment of nontraditional risk factors such as Lp(a), alongside detailed evaluation of GDMT use and adjunctive therapies targeting nontraditional pathways.

### 4.1 Limitations

Our systematic review and meta-analysis have several limitations that should be acknowledged. First, the predominance of observational cohort studies introduces inherent risks of residual confounding and selection bias that cannot be fully eliminated. Although we performed subgroup analyses according to ACS type, mean age, geographic region, economic classification, study period, and mortality follow-up duration to explore potential sources of heterogeneity, substantial heterogeneity remained unexplained. Variability in the definition of standard modifiable risk factors and SMuRFless status could theoretically contribute to between-study differences (14, 15, 32, 55); however, the included studies largely applied comparable definitions, making this an unlikely major source of heterogeneity. Nevertheless, the possibility that some patients classified as SMuRFless had unrecognized or previously undiagnosed traditional risk factors cannot be excluded. Finally, although we evaluated longer-term mortality outcomes, data for 2-, 3-, and 5-year mortality were limited, with only three studies contributing to each of these follow-up periods, thereby reducing the precision and generalizability of these estimates. Finally, given the predominantly observational nature of the included evidence, these associations should not be interpreted as demonstrating a causal effect of the SMuRFless phenotype itself.

## 5 Conclusions

This meta-analysis provides an updated examination of the clinical outcomes, procedural interventions, management disparities, and coronary vessel distribution in SMuRF-less ACS patients, a subgroup characterized by the absence of traditional cardiovascular risk factors such as hypertension, diabetes, dyslipidemia, and cigarette smoking. Despite a lower burden of conventional risk factors and broadly comparable rates of GDMT and PCI/thrombolysis, SMuRFless patients had significantly higher observed rates of early adverse outcomes, including in-hospital mortality, cardiogenic shock, and cardiac death. Coronary anatomy also differed, with lower rates of multivessel disease, LCX involvement, and combined RCA/LCX involvement, but more frequent LAD and LMCA involvement. Underutilization of ACEi/ARBs was also observed. These findings highlight the unique challenges and management gaps in SMuRF-less ACS and support further research into tailored clinical pathways, underlying pathophysiological mechanisms, and novel risk factors relevant to this population.

## Data Availability

The original contributions presented in the study are included in the article/Supplementary Material, further inquiries can be directed to the corresponding author/s.

## References

[1] Mechanic OJ Gavin M Grossman SA. Acute myocardial infarction. In: StatPearls. Treasure Island (FL): StatPearls Publishing (2025). Available online at: http://www.ncbi.nlm.nih.gov/books/NBK459269/ (Accessed April 2, 2025).

[2] Kumar A Cannon CP. Acute coronary syndromes: diagnosis and management, part I. Mayo Clin Proc. (2009) 84(10):917–38. 10.4065/84.10.917 19797781 PMC2755812

[3] Einarson TR Acs A Ludwig C Panton UH. Prevalence of cardiovascular disease in type 2 diabetes: a systematic literature review of scientific evidence from across the world in 2007–2017. Cardiovasc Diabetol. (2018) 17(1):83. 10.1186/s12933-018-0728-6 29884191 PMC5994068

[4] Silverman MG Ference BA Im K Wiviott SD Giugliano RP Grundy SM. Association between lowering LDL-C and cardiovascular risk reduction among different therapeutic interventions: a systematic review and meta-analysis. JAMA. (2016) 316(12):1289–97. 10.1001/jama.2016.13985 27673306

[5] Fuchs FD Whelton PK. High blood pressure and cardiovascular disease. Hypertension. (2020) 75(2):285–92. 10.1161/HYPERTENSIONAHA.119.14240 31865786 PMC10243231

[6] Wilson PWF D’Agostino RB Levy D Belanger AM Silbershatz H Kannel WB. Prediction of coronary heart disease using risk factor categories. Circulation. (1998) 97(18):1837–47. 10.1161/01.CIR.97.18.1837 9603539

[7] Dawber TR Moore FE Mann GV. II. Coronary heart disease in the framingham study. Am J Public Health Nations Health. (1957) 47(4_Pt_2):4–24. 10.2105/AJPH.47.4_Pt_2.4 13411327 PMC1550985

[8] Chunawala ZS Caughey MC Bhatt DL Hendrickson M Arora S Bangalore S. Mortality in patients hospitalized with acute myocardial infarction without standard modifiable risk factors: the ARIC study community surveillance. J Am Heart Assoc. (2023) 12(13):e027851. 10.1161/JAHA.122.027851 37382152 PMC10356095

[9] Gong W Yan Y Liu J Wang X Zheng W Que B. In-hospital mortality and treatment in patients with acute coronary syndrome with and without standard modifiable cardiovascular risk factors: findings from the CCC-ACS project. J Am Heart Assoc. (2024) 13(19):e029252. 10.1161/JAHA.122.029252 39291502 PMC11681477

[10] Vernon ST Coffey S Bhindi R Soo Hoo SY Nelson GI Ward MR. Increasing proportion of ST elevation myocardial infarction patients with coronary atherosclerosis poorly explained by standard modifiable risk factors. Eur J Prev Cardiol. (2017) 24(17):1824–30. 10.1177/2047487317720287 28703626

[11] Paul GJ Sankaran S Saminathan K Iliyas M Sethupathy S Saravanan S. Outcomes of ST segment elevation myocardial infarction without standard modifiable cardiovascular risk factors—newer insights from a prospective registry in India. Glob Heart. (2023) 18(1):13. 10.5334/gh.1189 36936250 PMC10022543

[12] Shamaki GR Safiriyu I Kesiena O Mbachi C Anyanwu M Zahid S. Prevalence and outcomes in STEMI patients without standard modifiable cardiovascular risk factors: a national inpatient sample analysis. Curr Probl Cardiol. (2022) 47(11):101343. 10.1016/j.cpcardiol.2022.101343 35934021

[13] Yaginuma H Saito Y Goto H Asada K Shiko Y Sato T. Impact of cancer, inflammation, and no standard risk factors in patients with myocardial infarction. JACC Asia. (2024) 4(7):507–16. 10.1016/j.jacasi.2024.03.008 39101117 PMC11291396

[14] Sheikh S Peerwani G Hanif B Virani S. Clinical characteristics, management, and 5-year survival compared between no standard modifiable risk factor (SMuRFless) and ≥1 SMuRF ACS cases: an analysis of 15,051 cases from Pakistan. BMC Cardiovasc Disord. (2023) 23(1):320. 10.1186/s12872-023-03355-z 37355597 PMC10290799

[15] Iwata J Inohara T Shiraishi Y Nakamaru R Niimi N Ueda I. Standard modifiable cardiovascular risk factors in patients with acute coronary syndrome: a report from multicenter percutaneous coronary intervention registry. J Cardiol. (2023) 81(6):571–6. 10.1016/j.jjcc.2023.01.009 36758671

[16] Kong G Chin YH Chong B Goh RSJ Lim OZH Ng CH. Higher mortality in acute coronary syndrome patients without standard modifiable risk factors: results from a global meta-analysis of 1,285,722 patients. Int J Cardiol. (2023) 371:432–40. 10.1016/j.ijcard.2022.09.062 36179904

[17] Page MJ McKenzie JE Bossuyt PM Boutron I Hoffmann TC Mulrow CD. The PRISMA 2020 statement: an updated guideline for reporting systematic reviews. Br Med J. (2021) 372:n71. 10.1136/bmj.n71 PMC8005924 33782057

[18] Schulman S Kearon C. Definition of major bleeding in clinical investigations of antihemostatic medicinal products in non-surgical patients. J Thromb Haemostasis. (2005) 3(4):692–4. 10.1111/j.1538-7836.2005.01204.x 15842354

[19] Munn Z Moola S Riitano D Lisy K. The development of a critical appraisal tool for use in systematic reviews: addressing questions of prevalence. Int J Health Policy Manag. (2014) 3(3):123–8. 10.15171/ijhpm.2014.71 25197676 PMC4154549

[20] Lin L Chu H. Meta-analysis of proportions using generalized linear mixed models. Epidemiology. (2020) 31(5):713–7. 10.1097/EDE.0000000000001232 32657954 PMC7398826

[21] Schwarzer G. Meta: General Package for Meta-Analysis. (2025). Available online at: https://cran.r-project.org/web/packages/meta/index.html (Accessed April 2, 2025).

[22] Van Aert RCM Jackson D. A new justification of the Hartung-Knapp method for random-effects meta-analysis based on weighted least squares regression. Res Synth Methods. (2019) 10(4):515–27. 10.1002/jrsm.1356 31111673 PMC6973024

[23] Egger M Smith GD Schneider M Minder C. Bias in meta-analysis detected by a simple, graphical test. Br Med J. (1997) 315(7109):629–34. 10.1136/bmj.315.7109.629 PMC2127453 9310563

[24] Higgins JPT Thomas J Chandler J Cumpston M Li T Page MJ. Cochrane Handbook for Systematic Reviews of Interventions Version. Amsterdam: Elsevier (2024), Vol. 6, No. 5.

[25] Lee KS Zhang JJY Nga VDW Ng CH Tai BC Higgins JPT. Tenets for the proper conduct and use of meta-analyses: a practical guide for neurosurgeons. World Neurosurg. (2022) 161:291–302.e1. 10.1016/j.wneu.2021.09.034 35505547

[26] McKenzie JE Veroniki AA. A brief note on the random-effects meta-analysis model and its relationship to other models. J Clin Epidemiol. (2024) 174:111492. 10.1016/j.jclinepi.2024.111492 39098563

[27] DerSimonian R Laird N. Meta-analysis in clinical trials. Control Clin Trials. (1986) 7(3):177–88. 10.1016/0197-2456(86)90046-2 3802833

[28] Fletcher J. What is heterogeneity and is it important?. BMJ(2007) 334:94–6. 10.1136/bmj.39057.406644.68 17218716 PMC1767262

[29] Khot UN Khot MB Bajzer CT Sapp SK Ohman EM Brener SJ. Prevalence of conventional risk factors in patients with coronary heart disease. JAMA. (2003) 290(7):898–904. 10.1001/jama.290.7.898 12928466

[30] Figtree GA Vernon ST Hadziosmanovic N Sundström J Alfredsson J Arnott C. Mortality in STEMI patients without standard modifiable risk factors: a sex-disaggregated analysis of SWEDEHEART registry data. Lancet. (2021) 397(10279):1085–94. 10.1016/S0140-6736(21)00272-5 33711294

[31] Vernon ST Coffey S D’Souza M Chow CK Kilian J Hyun K. ST-segment-elevation myocardial infarction (STEMI) patients without standard modifiable cardiovascular risk factors-how common are they, and what are their outcomes? J Am Heart Assoc. (2019) 8(21):e013296. 10.1161/JAHA.119.013296 31672080 PMC6898813

[32] Hammoudeh A Aldalal’ah M’ Smadi E Alrishoud D Alomari A Alkhawaldeh M. Absence of standard modifiable risk factors in Middle Eastern patients with atherosclerotic cardiovascular disease. The Jordan absence of standard modifiable risk factors (SMuRF-less) study. Vasc Health Risk Manag. (2025) 21:39–50. 10.2147/VHRM.S499355 39931041 PMC11807772

[33] Gallone G Baldetti L Pagnesi M Latib A Colombo A Libby P. Medical therapy for long-term prevention of atherothrombosis following an acute coronary syndrome: JACC state-of-the-art review. J Am Coll Cardiol. (2018) 72(23):2886–903. 10.1016/j.jacc.2018.09.052 30522652

[34] Levine GN Bates ER Bittl JA Brindis RG Fihn SD Fleisher LA. 2016 ACC/AHA guideline focused update on duration of dual antiplatelet therapy in patients with coronary artery disease. J Am Coll Cardiol. (2016) 68(10):1082–115. 10.1016/j.jacc.2016.03.513 27036918

[35] Byrne RA Rossello X Coughlan JJ Barbato E Berry C Chieffo A. 2023 ESC guidelines for the management of acute coronary syndromes: developed by the task force on the management of acute coronary syndromes of the European Society of Cardiology (ESC). Eur Heart J. (2023) 44(38):3720–826. 10.1093/eurheartj/ehad191 37622654

[36] Lawton JS Tamis-Holland JE Bangalore S Bates ER Beckie TM Bischoff JM. 2021 ACC/AHA/SCAI guideline for coronary artery revascularization: a report of the American College of Cardiology/American Heart Association joint committee on clinical practice guidelines. J Am Coll Cardiol. (2022) 79(2):e21–129. 10.1016/j.jacc.2021.09.006 34895950

[37] Palmerini T Bruno AG Gasparini M Rizzello G Kim H-S Kang J. Reduced mortality with antiplatelet therapy deescalation after percutaneous coronary intervention in acute coronary syndromes: a meta-analysis. Circ Cardiovasc Interv. (2022) 15(11):906–14. 10.1161/CIRCINTERVENTIONS.122.012245 36378738

[38] Sun B Liang C Sun P Aa G Li L Zhao L. Mortality after ST-segment–elevated myocardial infarction among patients with and without standard modifiable cardiovascular risk factors in China. Circ Popul Health Outcomes. (2026) 19(1):e012224. 10.1161/CIRCOUTCOMES.125.012224 41686017

[39] Cholesterol Treatment Trialists’ (CTT) Collaboration B. Efficacy and safety of more intensive lowering of LDL cholesterol: a meta-analysis of data from 170000 participants in 26 randomised trials. Lancet. (2010) 376(9753):1670–81. 10.1016/S0140-6736(10)61350-5 21067804 PMC2988224

[40] Goff DC Lloyd-Jones DM Bennett G Coady S D’Agostino RB Gibbons R. 2013 ACC/AHA guideline on the assessment of cardiovascular risk: a report of the American College of Cardiology/American Heart Association task force on practice guidelines. Circulation. (2014) 129(25 Suppl 2):S49–73. 10.1161/01.cir.0000437741.48606.98 24222018

[41] Akbar H Foth C Kahloon RA Mountfort S. Acute ST-segment elevation myocardial infarction (STEMI). In: StatPearls. Treasure Island (FL): StatPearls Publishing (2025). Available online at: http://www.ncbi.nlm.nih.gov/books/NBK532281/ (Accessed April 2, 2025). 30335314

[42] Nordlund D Heiberg E Carlsson M Fründ E-T Hoffmann P Koul S. Extent of myocardium at risk for left anterior descending artery, right coronary artery, and left circumflex artery occlusion depicted by contrast-enhanced steady state free precession and T2-weighted short tau inversion recovery magnetic resonance imaging. Circ Cardiovasc Imaging. (2016) 9(7):e004376. 10.1161/CIRCIMAGING.115.004376 27412659

[43] Ishii Y Langberg J Rosborough K Mikawa T. Endothelial cell lineages of the heart. Cell Tissue Res. (2009) 335(1):67–73. 10.1007/s00441-008-0663-z 18682987 PMC2729171

[44] Moon SH Byun JH Kim JW Kim SH Kim KN Jung JJ. Clinical usefulness of the angle between left main coronary artery and left anterior descending coronary artery for the evaluation of obstructive coronary artery disease. PLoS One. (2018) 13(9):e0202249. 10.1371/journal.pone.0202249 30212455 PMC6136703

[45] Miranda E Sousa LC António CC Castro CF Pinto SIS. Role of the left coronary artery geometry configuration in atherosusceptibility: CFD simulations considering sPTT model for blood. Comput Methods Biomech Biomed Eng. (2021) 24(13):1488–503. 10.1080/10255842.2021.1894555 33661071

[46] Entezarjou A Mohammad MA Andell P Koul S. Culprit vessel: impact on short-term and long-term prognosis in patients with ST-elevation myocardial infarction. Open Heart. (2018) 5(2):e000852. 10.1136/openhrt-2018-000852 30228908 PMC6135450

[47] Gorenek B Blomström Lundqvist C Brugada Terradellas J Camm AJ Hindricks G Huber K. Cardiac arrhythmias in acute coronary syndromes: position paper from the joint EHRA, ACCA, and EAPCI task force. EuroIntervention. (2015) 10(9):1095–108. 10.4244/EIJY14M08_19 25169596

[48] Rene AG Généreux P Ezekowitz M Kirtane AJ Xu K Mehran R. Impact of atrial fibrillation in patients with ST-elevation myocardial infarction treated with percutaneous coronary intervention [from the HORIZONS-AMI (harmonizing outcomes with revascularization and stents in acute myocardial infarction) trial]. Am J Cardiol. (2014) 113(2):236–42. 10.1016/j.amjcard.2013.09.016 24176066

[49] Lopes RD Elliott LE White HD Hochman JS Van de Werf F Ardissino D. Antithrombotic therapy and outcomes of patients with atrial fibrillation following primary percutaneous coronary intervention: results from the APEX-AMI trial. Eur Heart J. (2009) 30(16):2019–28. 10.1093/eurheartj/ehp213 19502623 PMC2764954

[50] Ball J Dinh DT Brennan A Ajani A Clark DJ Freeman M. Prevalence and outcomes of patients with SMuRF-less acute coronary syndrome undergoing percutaneous coronary intervention. Open Heart. (2024) 11(1):e002733. 10.1136/openhrt-2024-002733 38843905 PMC11163639

[51] Figtree GA Vernon ST Harmer JA Gray MP Arnott C Bachour E. Clinical pathway for coronary atherosclerosis in patients without conventional modifiable risk factors: JACC state-of-the-art review. J Am Coll Cardiol. (2023) 82(13):1343–59. 10.1016/j.jacc.2023.06.045 37730292 PMC10522922

[52] Vernon ST Hansen T Kott KA Yang JY O’Sullivan JF Figtree GA. Utilizing *state-of-the-art* “omics” technology and bioinformatics to identify new biological mechanisms and biomarkers for coronary artery disease. Microcirculation. (2019) 26(2):e12488. 10.1111/micc.12488 29956866

[53] Piepoli MF Hoes AW Agewall S Albus C Brotons C Catapano AL. 2016 European guidelines on cardiovascular disease prevention in clinical practice: the sixth joint task force of the European Society of Cardiology and other societies on cardiovascular disease prevention in clinical practice (constituted by representatives of 10 societies and by invited experts)Developed with the special contribution of the European association for cardiovascular prevention & rehabilitation (EACPR). Eur Heart J. (2016) 37(29):2315–81. 10.1093/eurheartj/ehw106 27222591 PMC4986030

[54] Playford D Hamilton-Craig C Dwivedi G Figtree G. Examining the potential for coronary artery calcium (CAC) scoring for individuals at low cardiovascular risk. Heart Lung Circ. (2021) 30(12):1819–28. 10.1016/j.hlc.2021.04.026 34332891

[55] Kong G Chew NWS Ng CH Chin YH Lim OZH Ambhore A. Prognostic outcomes in acute myocardial infarction patients without standard modifiable risk factors: a multiethnic study of 8,680 Asian patients. Front Cardiovasc Med. (2022) 9:869168. 10.3389/fcvm.2022.869168 35425823 PMC9001931

