# Supplementary material for: An updated meta-analysis of clinical outcomes, interventions, and treatment approaches in SMuRF vs. SMuRFless: evidence from 2.6 million ACS patients
Source: Front Cardiovasc Med. 2026 Oct 29;13:1931296. doi: 10.3389/fcvm.2026.1931296 (PMC13754722; doi:10.3389/fcvm.2026.1931296)
Supplement: Supplementary file 1 [file Datasheet1.docx]

**An Updated Meta-Analysis of Clinical Outcomes, Interventions, and Treatment Approaches in SMuRF vs. SMuRF-less: Evidence from 2.6 million ACS Patients**

**Supplementary File**

**Supplementary Table 1.** Complete Search Strategy

(“Acute Coronary Syndrome”[Mesh] OR “Myocardial Infarction”[Mesh] OR “Acute Coronary Syndrome”[TIAB] OR “ACS”[TIAB] OR “Myocardial Infarction”[TIAB] OR “Heart Attack”[TIAB])

AND

((“SMuRF”[TIAB] OR “SMuRF-less”[TIAB] OR “Risk Factors”[Mesh] OR “Cardiovascular Risk”[TIAB] OR “Traditional Risk Factors”[TIAB] OR “Modifiable Risk Factors”[TIAB])

OR

(“Mortality”[Mesh] OR “In-Hospital Mortality”[TIAB] OR “Cardiac Death”[TIAB] OR “Cardiogenic Shock”[TIAB] OR “Clinical Outcomes”[TIAB])

OR

(“Coronary Artery Bypass”[Mesh] OR “Percutaneous Coronary Intervention”[Mesh] OR “CABG”[TIAB] OR “PCI”[TIAB] OR “Revascularization”[TIAB])

OR

(“Angiotensin-Converting Enzyme Inhibitors”[Mesh] OR “ACE Inhibitors”[TIAB] OR “Angiotensin Receptor Blockers”[Mesh] OR “ARBs”[TIAB] OR “Beta-Blockers”[TIAB] OR “Statins”[TIAB] OR “Guideline-Directed Medical Therapy”[TIAB] OR “GDMT”[TIAB]))

**Supplementary Table 2.** Databases Search Strategy

| **Database** | **Results retrieved** | **Search** |
| --- | --- | --- |
| Pubmed | 64 | ( "Acute Coronary Syndrome"[Mesh] OR "Acute Coronary Syndrome"[tiab] OR ACS[tiab] OR "Myocardial Infarction"[Mesh] OR "Myocardial Infarction"[tiab] ) AND ( SMuRF[tiab] OR "SMuRF-positive"[tiab] OR "SMuRF-positive"[tiab] OR "SMuRF less"[tiab] OR "SMuRF-less"[tiab] OR SMuRFless[tiab]) |
| Scopus | 97 | ( "Acute Coronary Syndrome" OR "Myocardial Infarction" OR ACS ) AND ( SMuRF OR "standard modifiable risk factor*" ) |
| Clinical Trials | 2 | AREA[ConditionSearch](Acute Coronary Syndrome) AND AREA[InterventionSearch](Standard Modifiable Risk Factors) |
| Cochrane | 52 | ("acute coronary syndrome"):ti,ab,kw AND (Standard Modifiable Risk Factors):ti,ab,kwAND  (SMuRF OR 'standard modifiable risk factor*') |

**Supplementary Table 3.** Joanna Brigg’s Institute Critical Appraisal Quality Assessment

| **Study** | **Q1** | **Q2** | **Q3** | **Q4** | **Q5** | **Q6** | **Q7** | **Q8** | **Q9** | **Q10** | **Q11** | **% yes** | **Risk** |
| --- | --- | --- | --- | --- | --- | --- | --- | --- | --- | --- | --- | --- | --- |
| Alberty (2017) | ✔️ | ✔️ | ✔️ | ✔️ | ✔️ | N/A | ✔️ | N/A | N/A | N/A | ✔️ | 100% | Low |
| Figtree (2021) | ✔️ | ✔️ | ✔️ | ✔️ | ✔️ | ✔️ | ✔️ | ✔️ | ✔️ | ✔️ | ✔️ | 100% | Low |
| Figtree (2022) | ✔️ | ✔️ | ✔️ | ✔️ | ✔️ | ✔️ | ✔️ | ✔️ | ✔️ | ✔️ | ✔️ | 100% | Low |
| Gonzalez-Pacheco (2014) | ✔️ | ✔️ | ✔️ | ✔️ | **X** | N/A | ✔️ | N/A | N/A | N/A | ✔️ | 86% | Low |
| Khot  (2003) | ✔️ | ✔️ | ✔️ | ✔️ | ✔️ | N/A | ✔️ | N/A | N/A | N/A | ✔️ | 100% | Low |
| Kong (2021) | ✔️ | ✔️ | ✔️ | ✔️ | ✔️ | ✔️ | ✔️ | ✔️ | ✔️ | **X** | ✔️ | 91% | Low |
| Li  (2022) | ✔️ | ✔️ | ✔️ | ✔️ | ✔️ | ✔️ | ✔️ | ✔️ | ✔️ | ✔️ | ✔️ | 100% | Low |
| Papazoglou (2022) | ✔️ | ✔️ | ✔️ | ✔️ | ✔️ | ✔️ | ✔️ | ✔️ | ✔️ | **X** | ✔️ | 91% | Low |
| Roe  (2007) | ✔️ | ✔️ | ✔️ | ✔️ | ✔️ | ✔️ | ✔️ | ✔️ | ✔️ | **X** | ✔️ | 91% | Low |
| Shamaki (2022) | ✔️ | ✔️ | ✔️ | ✔️ | ✔️ | ✔️ | ✔️ | ✔️ | ✔️ | **X** | ✔️ | 91% | Low |
| Shrestha (2022) | ✔️ | ✔️ | ✔️ | ✔️ | ✔️ | ✔️ | ✔️ | ✔️ | ✔️ | **X** | ✔️ | 91% | Low |
| Vernon (2020) | ✔️ | ✔️ | ✔️ | ✔️ | ✔️ | ✔️ | ✔️ | ✔️ | ✔️ | **X** | ✔️ | 91% | Low |
| Vernon (2019) | ✔️ | ✔️ | ✔️ | ✔️ | ✔️ | ✔️ | ✔️ | ✔️ | ✔️ | ✔️ | ✔️ | 100% | Low |
| Wang (2015) | ✔️ | ✔️ | ✔️ | ✔️ | ✔️ | ✔️ | ✔️ | ✔️ | ✔️ | **X** | ✔️ | 91% | Low |
| Yamamoto (2022) | ✔️ | ✔️ | ✔️ | ✔️ | ✔️ | ✔️ | ✔️ | ✔️ | ✔️ | ✔️ | ✔️ | 100% | Low |
| Yamamoto (2022) | ✔️ | ✔️ | ✔️ | ✔️ | ✔️ | N/A | ✔️ | N/A | N/A | N/A | ✔️ | 100% | Low |
| Anderson (2023) | ✔️ | ✔️ | ✔️ | ✔️ | ✔️ | ✔️ | ✔️ | ✔️ | ✔️ | ✔️ | ✔️ | 100% | Low |
| Paul (2023) | ✔️ | ✔️ | ✔️ | ✔️ | ✔️ | ✔️ | ✔️ | ✔️ | ✔️ | ✔️ | ✔️ | 100% | Low |
| Saito (2023) | ✔️ | ✔️ | ✔️ | ✔️ | X | ✔️ | ✔️ | ✔️ | ✔️ | X | ✔️ | 82% | Low |
| Moledena (2022) | ✔️ | ✔️ | ✔️ | ✔️ | ✔️ | ✔️ | ✔️ | ✔️ | ✔️ | ✔️ | ✔️ | 100% | Low |
| Kelly (2023) | ✔️ | ✔️ | ✔️ | ✔️ | ✔️ | ✔️ | ✔️ | ✔️ | ✔️ | X | ✔️ | 91% | Low |
| Mazhar (2022) | ✔️ | ✔️ | ✔️ | ✔️ | ✔️ | ✔️ | ✔️ | ✔️ | ✔️ | ✔️ | ✔️ | 100% | Low |
| Jang (2023) | ✔️ | ✔️ | ✔️ | ✔️ | ✔️ | ✔️ | ✔️ | ✔️ | ✔️ | X | ✔️ | 91% | Low |
| Iwata (2023) | ✔️ | ✔️ | ✔️ | ✔️ | ✔️ | ✔️ | ✔️ | ✔️ | ✔️ | X | ✔️ | 91% | Low |
| Gladys (2024) | ✔️ | ✔️ | ✔️ | ✔️ | ✔️ | ✔️ | ✔️ | ✔️ | ✔️ | ✔️ | ✔️ | 100% | Low |
| Zao (2023) | ✔️ | ✔️ | ✔️ | ✔️ | ✔️ | ✔️ | ✔️ | ✔️ | ✔️ | ✔️ | ✔️ | 100% | Low |
| Weight (2023) | ✔️ | ✔️ | ✔️ | ✔️ | ✔️ | ✔️ | ✔️ | ✔️ | ✔️ | ✔️ | ✔️ | 100% | Low |
| Suzuki (2022) | ✔️ | ✔️ | ✔️ | ✔️ | ✔️ | ✔️ | ✔️ | ✔️ | ✔️ | X | ✔️ | 91% | Low |
| Singh (2024) | ✔️ | ✔️ | ✔️ | ✔️ | ✔️ | ✔️ | ✔️ | ✔️ | ✔️ | ✔️ | ✔️ | 100% | Low |
| Sia (2022) | ✔️ | ✔️ | ✔️ | ✔️ | ✔️ | ✔️ | ✔️ | ✔️ | ✔️ | ✔️ | ✔️ | 100% | Low |
| Sheikh (2023) | ✔️ | ✔️ | ✔️ | ✔️ | ✔️ | ✔️ | ✔️ | ✔️ | ✔️ | X | ✔️ | 91% | Low |
